# Enterprise marketing strategy using big data mining technology combined with XGBoost model in the new economic era

**DOI:** 10.1371/journal.pone.0285506

**Published:** 2023-06-05

**Authors:** Huijun Chen

**Affiliations:** Wuhan City Polytechnic, Wuhan City, China; Sejong University, REPUBLIC OF KOREA

## Abstract

The technological development in the new economic era has brought challenges to enterprises. Enterprises need to use massive and effective consumption information to provide customers with high-quality customized services. Big data technology has strong mining ability. The relevant theories of computer data mining technology are summarized to optimize the marketing strategy of enterprises. The application of data mining in precision marketing services is analyzed. Extreme Gradient Boosting (XGBoost) has shown strong advantages in machine learning algorithms. In order to help enterprises to analyze customer data quickly and accurately, the characteristics of XGBoost feedback are used to reverse the main factors that can affect customer activation cards, and effective analysis is carried out for these factors. The data obtained from the analysis points out the direction of effective marketing for potential customers to be activated. Finally, the performance of XGBoost is compared with the other three methods. The characteristics that affect the top 7 prediction results are tested for differences. The results show that: (1) the accuracy and recall rate of the proposed model are higher than other algorithms, and the performance is the best. (2) The significance p values of the features included in the test are all less than 0.001. The data shows that there is a very significant difference between the proposed features and the results of activation or not. The contributions of this paper are mainly reflected in two aspects. 1. Four precision marketing strategies based on big data mining are designed to provide scientific support for enterprise decision-making. 2. The improvement of the connection rate and stickiness between enterprises and customers has played a huge driving role in overall customer marketing.

## 1 Introduction

The 21st century is a new era of digitalization. The whole of human society has entered the information digital society. The development of information society must rely on the development of advanced network computer technology. Internet technology enables enterprises to communicate with customers at zero distance and complete transactions on the network [1]. The explosive expansion of the Internet has made it a revolutionary technology in the new millennium. It has also brought blowout data growth, giving consumers and companies similar connectivity. The Internet and other computer technologies have gradually changed how companies provide market services. The new generation of network marketers and channel relationships have replaced traditional marketers. Meanwhile, the new technology also helps marketers effectively provide market supplies according to target customers’ needs and even helps customers design their own market supplies [2].

Customer information has become a crucial strategic resource and marketing tool in the rapidly changing environment. Managers need the latest information to make timely and high-quality decisions. Besides, companies can collect a lot of information with the explosion of information technology. In fact, currently, managers often get too much information, so the lack of information is not a problem. In this era of such massive information, how to use computer data mining technology to penetrate the information fog to find better-quality information is now the problem to be solved.

More and more industries and technical fields need big data analysis systems. The analysis system used to support the requirements of these scenarios faces roughly the same technical challenges [3]. ① The process of data analysis requires both historical information and real-time information. Big data technology can meet this demand. This technology has exploratory characteristics, which are convenient for follow-up tracking of historical data. ② Users may store massive historical data. Additionally, the data scale has a trend of continuous growth. It is necessary to introduce distributed storage systems to meet the requirements of reliability and scalability, and control costs. ③ The technology stack needs to combine streaming components, storage systems, computing components, etc. ④ The system has high operational and maintenance requirements. Complex big data architecture is difficult to maintain and control.

This exploration aims to analyze and study the current situation of customer management in financial enterprises and find out the current management deficiencies. It also aims to use the XGBoost algorithm to establish a stock customer activation prediction model, and rely on the model and customer relationship-related theories to develop and improve the stock customer activation management strategy. Based on the research on the relevant technical theory of data mining and the relevant theory of the XGBoost algorithm in the application of precision marketing, the original data of all users of a financial enterprise are taken as the analysis object. According to the characteristics of the industry market, the overall planning and key contents of the company’s precision marketing research are identified. Four precision marketing strategies based on big data mining have been designed, and successful cases of application of mining results have been displayed, providing scientific support for enterprises’ marketing decisions.

Firstly, this paper reviews the current research status, summarizes the literature review of existing data mining techniques used in enterprise marketing, organizes advantages and disadvantages, and makes improvements. Secondly, the research summarizes the introduction of relevant theories and models used, including computer data mining technology and the XGBoost algorithm model. Next, the model is designed. The training process and indicator design of the XGBoost algorithm are highlighted, laying a solid foundation for subsequent experiments. Finally, the results of the experiment are summarized. Section 1 is the detection of the XGBoost algorithm, which selects the optimal model by calculating evaluation indicators. Section 2 is an analysis of the impact of marketing strategies on customers. Strategies that can improve marketing methods are proposed by summarizing the impact of different enterprise natures on customer consumption and customer distribution.

## 2 Literature review

Currently, using machine learning (ML) to analyze marketing tools can help enterprises extract meaningful information from massive data. For example, chat robots can be adapted to assist salesmen in offering customer service and interacting with customers on social media. ML can be employed to process medical equipment data to enhance the understanding of the current situation of medical equipment marketing. In the research on the sales forecast of the physical retail industry, Yang et al. used a lot of feature engineering and models to build a combination model based on XGBoost and compared it with a single model. It is found that the combined model performs much better than the single XGBoost model [4]. Bi et al. built a data mining model based on clustering optimization random forest (RF) using the telecom customer data in Southeast Asia and achieved high accuracy and efficiency [5]. In the medical field, Gao et al. pointed out that using ML technology in neuroimaging can automatically classify schizophrenia patients and healthy control groups [6]. Lassau et al. adopted the integrated learning method XGBoost to predict the early death of hospitalized elderly patients with multiple organ dysfunction syndrome better to assist clinical decision-making and treatment [7]. Reddy et al. proposed a new deep learning method, namely an end-to-end multi-layer wavelet convolutional neural network, to diagnose heart disease. Many domestic scholars have also studied the classification of medical disputes [8]. Liu et al. applied the XGBoost algorithm to build a prediction model for dydrogesterone’s effectiveness and safety in treating threatened abortion [9].

The market of computer data mining tools is generally classified into general tools, comprehensive data mining tools, and rapidly developing tools for specific applications. By analyzing the current situation, Kar and Dwivedi objectively pointed out that people have entered the era of big data. Besides, human and material resources will become inaccessible production factors in the new era [10]. Hence, using data mining to master big data information is equal to possessing superior resources, which can improve production efficiency, and will greatly benefit the company’s economic output. Mittal et al. proposed another advantage of data mining technology on the basis of an in-depth study of the development status of data mining technology in various countries. This technology can better optimize government functions, comprehensively improve service quality, shorten project operation processes, and facilitate macro-control of the economy in a broader range. In this way, the public can better praise government services, improving citizen participation and satisfaction [11]. Currently, multiple national institutions for data mining research have also emerged. For example, the Data Mining Center of the Department of Statistics of the Renmin University of China and the Innovation and Training Center of the School of Management of the Fu Jen Catholic University of Taiwan has been engaged in data mining research in recent years. Among them, Wang and Huang conducted a more in-depth study on applying fuzzy methods in knowledge discovery [12]. Yang et al. also researched data cube algebra, discussing knowledge discovery of unstructured data and Web data mining [13].

Previous studies have shown that acquiring and processing a large amount of potential customer information based on the marketing model will gradually increase marketing consumption. The reduction of enterprise profits will greatly limit its long-term development. The research innovation is to collect, process, and analyze massive customer application and transaction data through data mining algorithms to make the characteristics of customers’ card activation and consumption behavior become regular. Moreover, XGBoost and other ML algorithms are applied to calculate and evaluate the characteristic factors affecting customer consumption. These factors help banks make more scientific and reasonable decisions on the management of existing customers. Meanwhile, combined with customer segmentation theory, customer life cycle, and other relevant theories, the stock customers are subdivided by using different consumer preferences of customers. In addition, more differentiated management strategies are adopted for customer groups with different consumer preferences, improving customer experience and promoting the frequency and amount of cards used to improve the value of stock customers.

## 3 Materials and methods

### 3.1 Computer data mining technology and related theories

**Fig 1:**
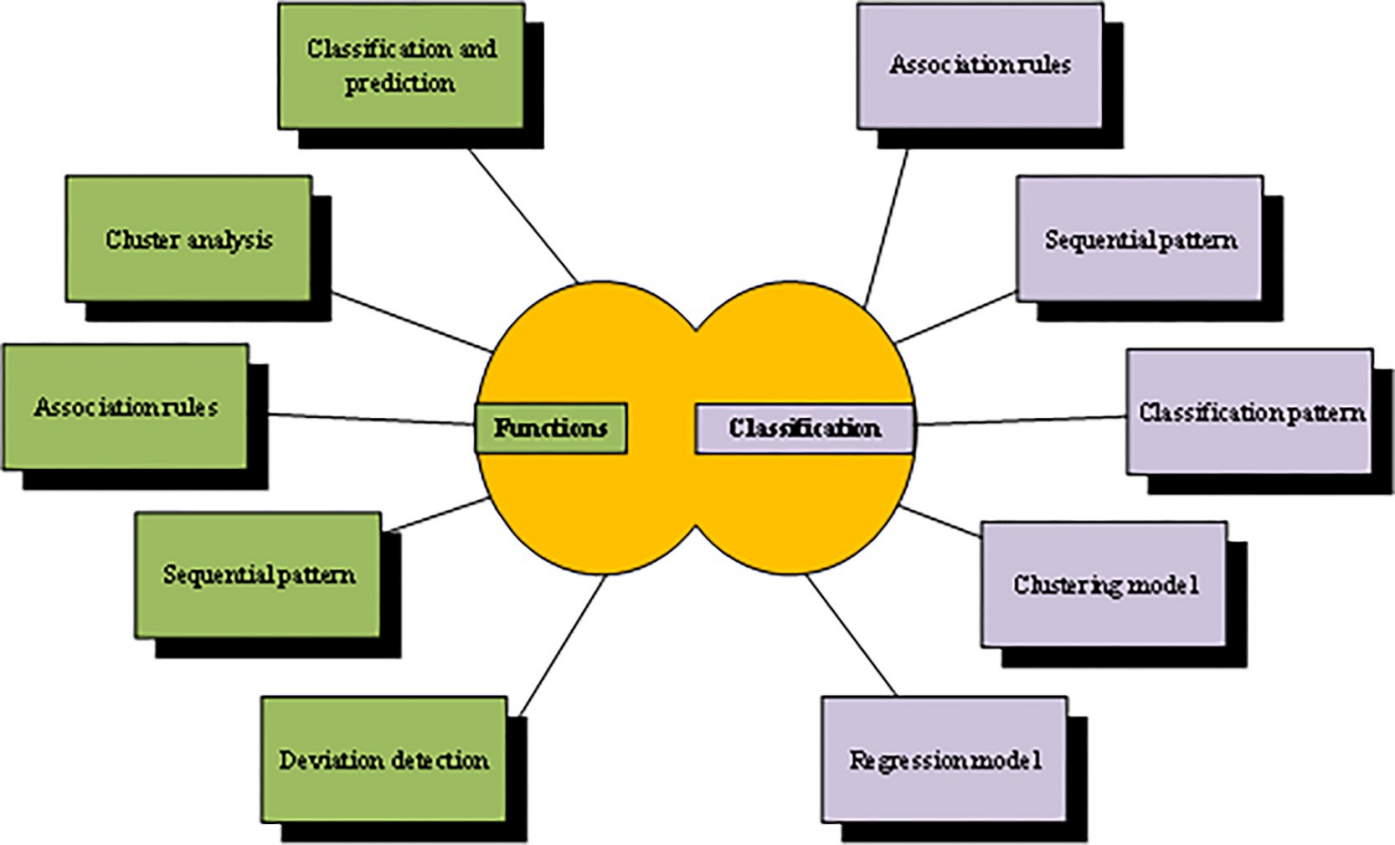
Functions and classification of computer data mining technology.

Data mining methods are based on mathematical theory, non-mathematical, deductive, and inductive [14]. Based on the historical level of research, they are the theoretical systems created by scholars and engineers in the database, Artificial Intelligence (AI), mathematical statistics, computer science, and other fields in the exploratory research process of data mining. The functions and classification of computer data mining technology [15] are shown in Fig 1:

Data needs more efficient and detailed processing to provide a basis for subsequent work, and data mining technology has helped mankind achieve this goal. Through classification, integration, analysis, and data comparison, the number of data has increased massively, and the data types are changing. The previous data processing methods are far from being able to complete this task, so the introduction of data mining technology has its inevitability, and the adopted algorithm is more complex. This technology is conducive to upgrading big data analysis [16]. The core algorithms and technologies used in data mining are the core part of the data mining platform. The algorithms and technologies closely related to data mining mainly include:

1. ML includes two learning methods: neural network and decision tree algorithm. The neural network is a kind of self-organization learning, and the decision tree algorithm is a combination of computer and AI. The purpose of the neural network algorithm is mainly to solve problems with high complexity. It comprises simple processing units and many parallel distributed processing units. Through learning experiences in daily life, the connection strength between these experiences is adjusted, and then its empirical principle is applied to practical problems. The decision tree is a common ML algorithm. It is a process of making decisions based on a tree graph. It is usually a process of continuous judgment to make a decision. This algorithm first needs to determine a classification-oriented model, classify the problems according to the branches, and gradually solve the data mining problem. The advantage of the decision tree algorithm is to display the whole judgment process, which is intuitive and easy to understand.
2. Statistics can support data mining technology, including sampling, prediction algorithm (regression), experience-based design, etc.
3. Decision support system. The system is based on management science, operational research, cybernetics, and behavioral science and uses computer, information, and simulation technology. It is an intelligent man-machine system that supports decision-making activities for semi-structured decision-making problems. The decision support system can provide data services for enterprises, including establishing decision objectives, establishing research issues, constructing or improving decision models, providing alternatives, and evaluating and selecting alternatives. This system can provide a series of scientific, effective, and reliable decision-making schemes for decision-makers through human-computer interaction (HCI) functions to realize scientific decision-making.
4. Data warehouse is a special form of database that can provide supporting data for enterprises’ decision-making at all levels. It is built for decision support and specific analysis process. The data warehouse can store a single data body and provide business process improvement, cost saving, and quality control for enterprises that need AI.

**Fig 2:**
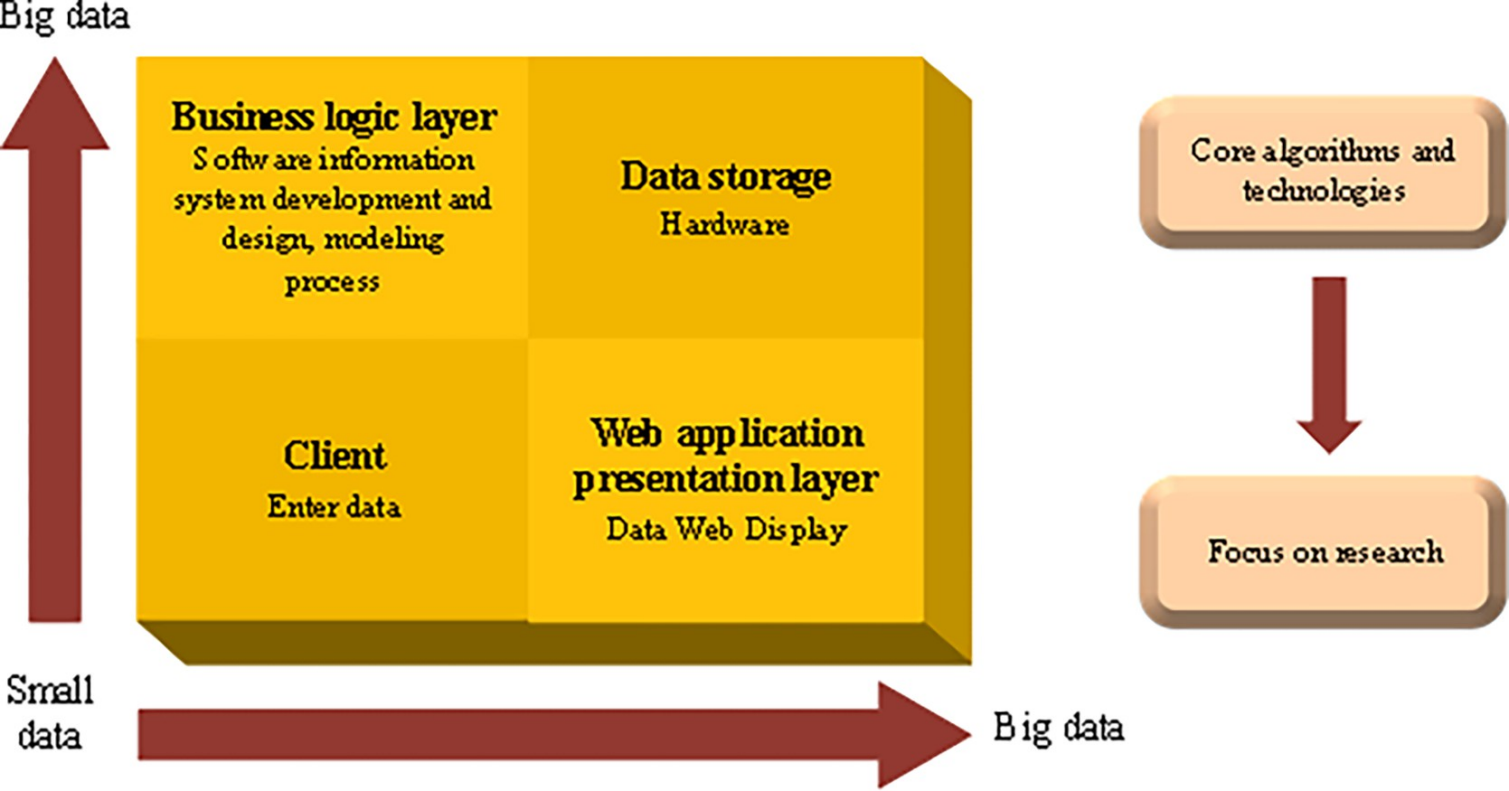
Schematic diagram of data information processing process.

In recent years, with the progress of decision support technology, data mining technology has attracted the attention of academic and business circles. Modern society is where a large amount of information data is generated every moment. Enterprises and markets convert these data into useful information and knowledge and apply them to operations. Customer demand analysis, market analysis, production control, etc., can strongly support enterprise decision-making. The data mining technology platform includes three main contents: algorithm and technology, data, and modeling ability [17]. Fig 2 displays the logical process of transferring data on the mining platform and then transforming them into information that can be used:

It reveals that the core algorithm and technology used in data mining is the core part of the data mining platform. The algorithms and technologies closely related to data mining mainly include ML, statistics, decision support systems, and data warehouses. Front-end tools mainly include reports, data analysis, queries, data mining, and various application development tools based on a data warehouse or data mart [18].

**Fig 3:**
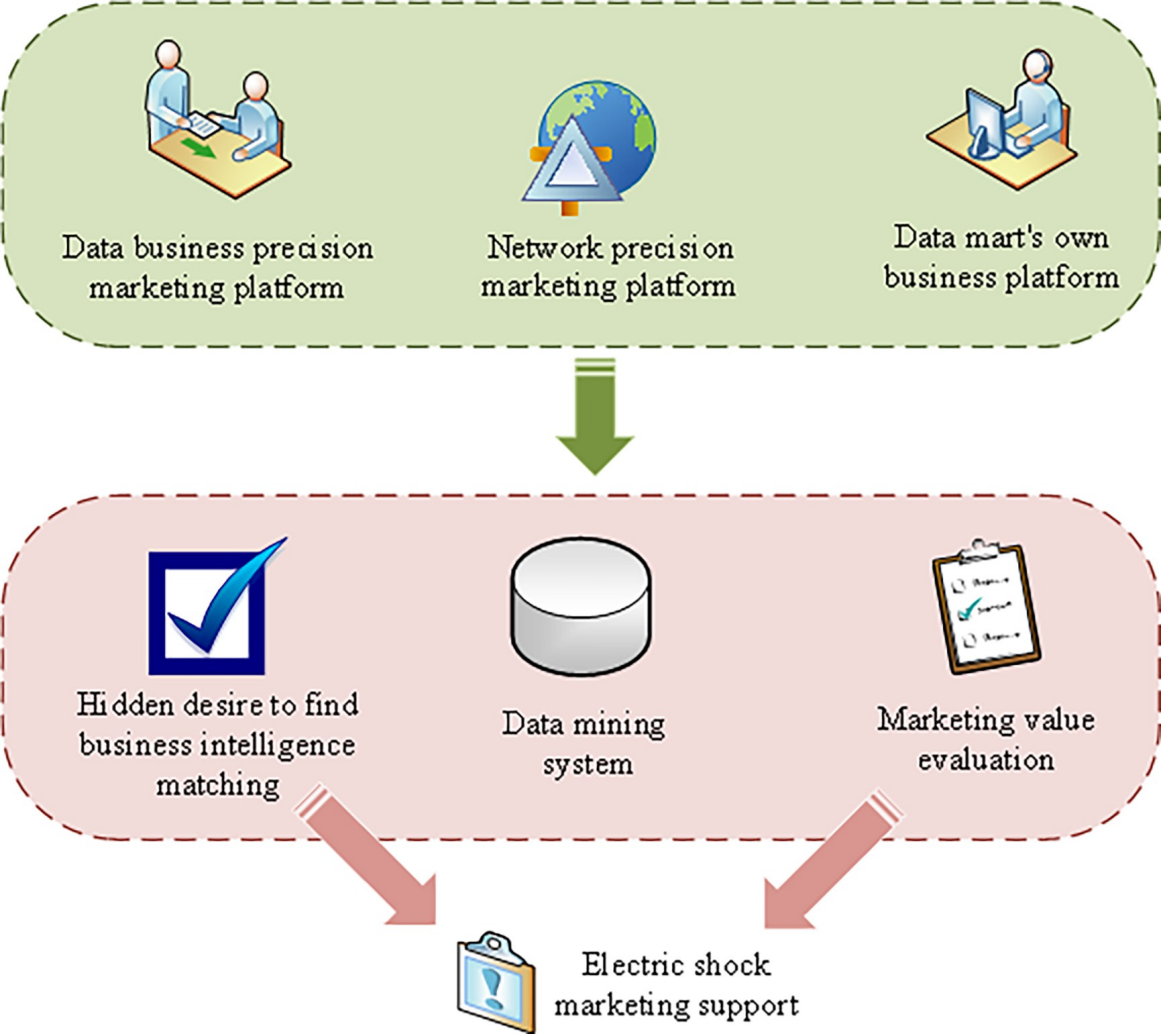
Data mining system model.

Generally, enterprises establish a complete data mining system based on the precision marketing system of big data mining. The overall design idea is aggregation–mining–operation—evaluation. Fig 3 presents the overall data mining system model.

Enterprise data collection is mainly classified into three aspects according to the different data sources: recruitment, business, and network. These data form a big data aggregation information base. Meanwhile, the information storage layer between and within each information source is also different. The network signaling data are mainly the user basic data dimension table, including terminal dimension table, area dimension table, music dimension table, and other dimension tables [19].

**Fig 4:**
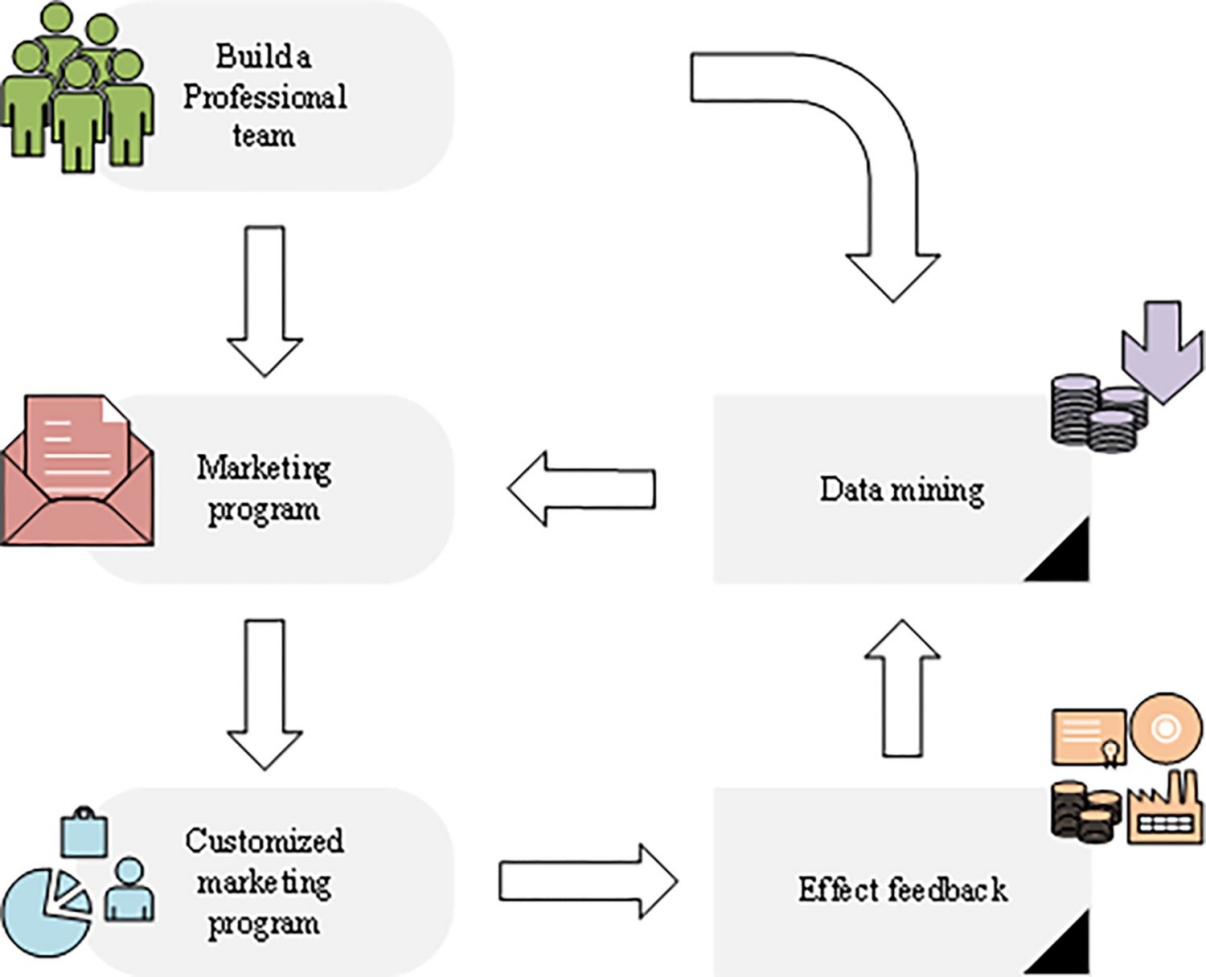
Marketing model based on data mining.

Based on the data of large databases, various complex calculation methods are used to analyze various data to sort out more valuable information data, which is data mining technology [20]. This technology has realized data transformation, so its value has been fully utilized. Based on the study of the issues and the analysis of the current situation of various departments of the enterprise, a new marketing process is initially proposed, as shown in Fig 4 [21]:

A new professional team has been established using the traditional marketing method. The conclusion of data mining is integrated into the traditional marketing plan. According to the customer performance in the marketing process, the customer manager’s subjective initiative is brought into play to provide users with flexible financial allocation schemes, and the final marketing results are fed back to the professional team [22].

### 3.2 XGBoost algorithm

**Fig 5:**
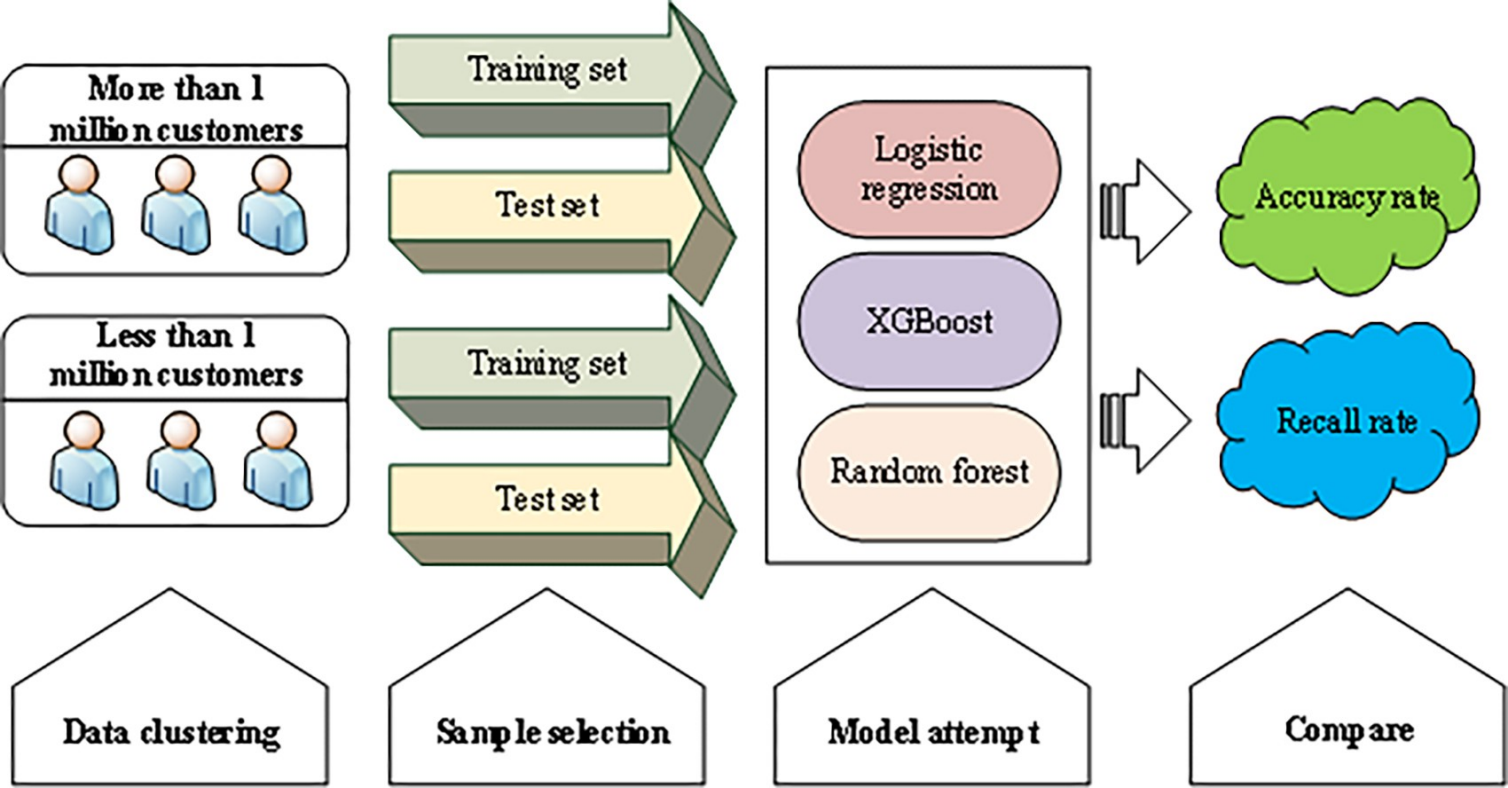
Attempt framework of the model.

In terms of model selection, this paper has tried various ML algorithms to predict and analyze Very Important Person (VIP) customer churn behavior. The overall idea of the model is shown in Fig 5:

The prediction effect of the same model is the same for different customer groups. According to the model’s performance, XGBoost is selected as the model algorithm. After selecting the XGBoost model, the grid search algorithm is adopted to optimize and adjust the parameters. The parameters that have the most important impact on the model are used as the starting point and trained according to the descending direction of the importance of the impact on the model. Before each training, the optimal solution obtained is fixed as input and iterated [23].

**Fig 6:**
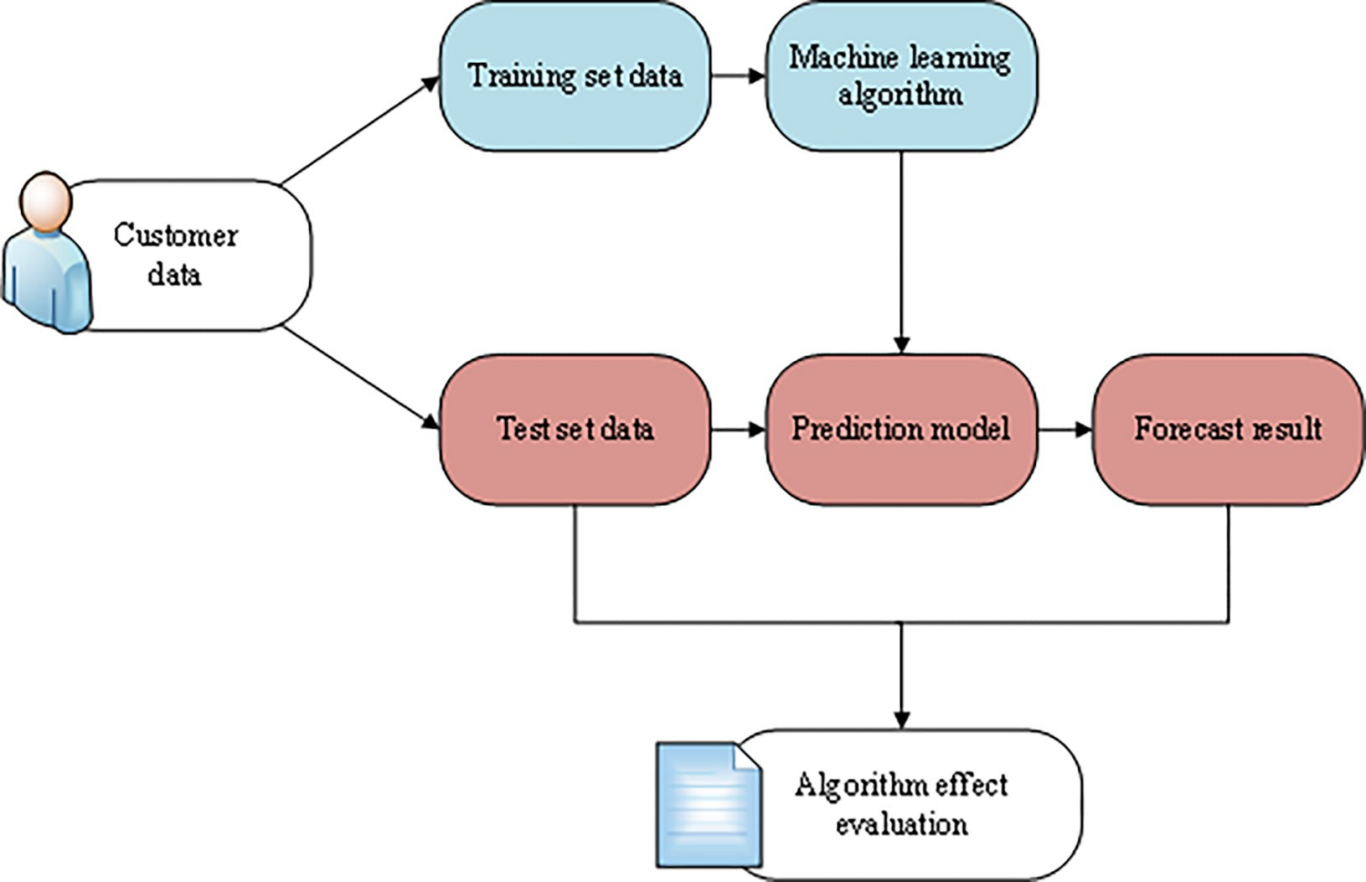
Training process of model construction.

**Fig 7:**
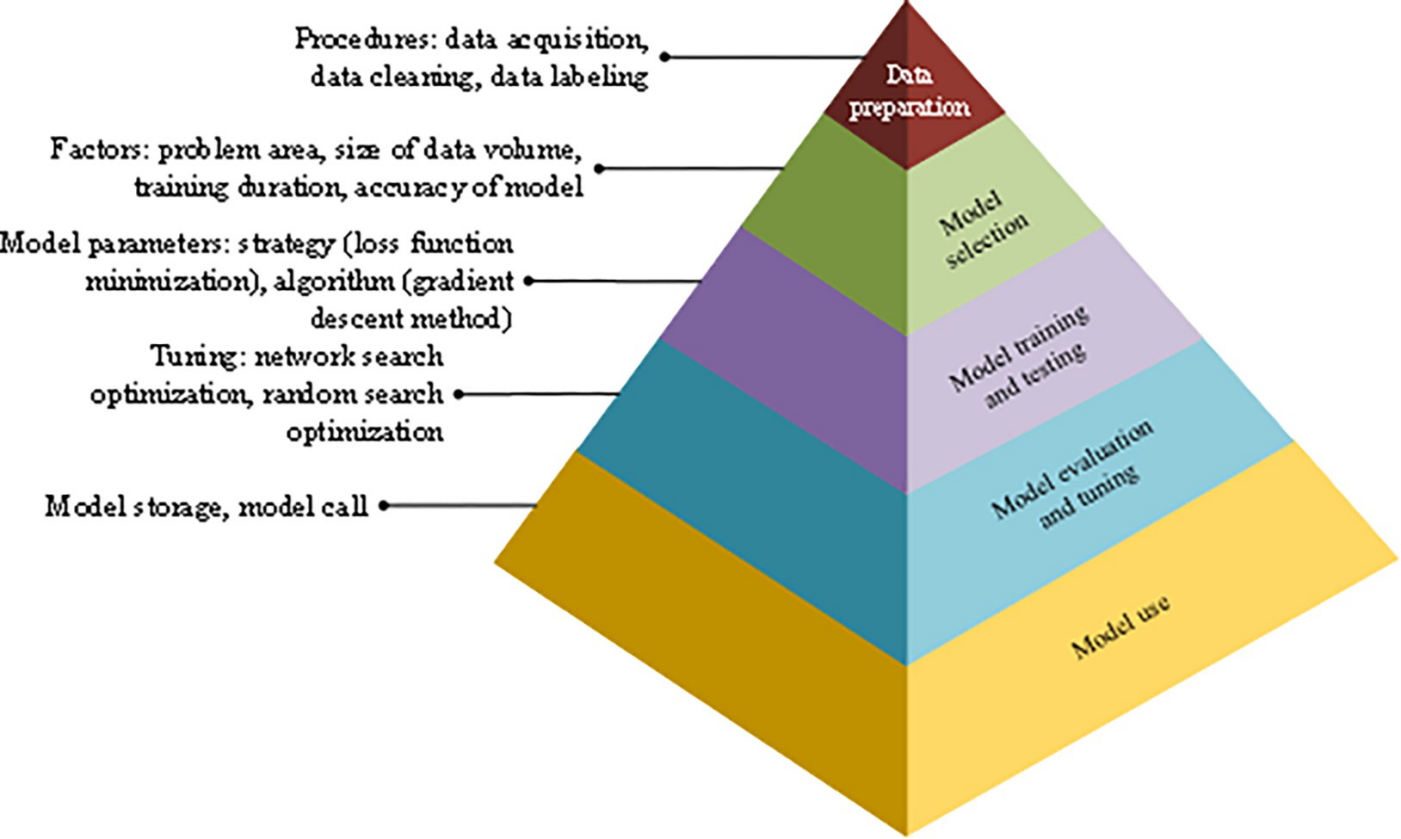
Construction process of XGBoost model.

After the algorithm of model construction is selected, the model is constructed according to the standard data mining process. After source data processing and feature engineering, the sample data set is divided into 70% of the training set and 30% of the test set. The data of the training set is input into the specified algorithm to build the activation model of the potential card. Additionally, the data of the test set is input into the built prediction model. Determine whether the user is a potential cooperative customer through the known relevant information, compare the test positive sample results and the prediction, and evaluate the effectiveness of its algorithm. The process of model training is shown in Fig 6:

XGBoost model results from ML and the learning process is called training. The model can be understood as a function: *y* = *f*(*x*). The parameters of the function are mainly learned from the training set. According to the type of output results, it can be divided into the regression model and classification model. If *y* is a numerical value, the model is a regression model. If *y* is a label, it is a classification model [24]. The model construction process generally includes data preparation, model selection, model training and testing, model evaluation and tuning, and model use, as shown in Fig 7.

The data preparation stage includes data collection, cleaning, and labeling. The model selection is closely related to the problem area, the size of the data volume, the training duration, the model accuracy, and other factors. As the basis of model training, data are usually classified into training and test sets [25]. The data set is divided into the training set and test set according to 7:3. Parameters of the data set are selected through cross-validation, as shown in Table 1:

**Table 1:** Selection of data set parameters.

Parameter	Value
Number of categories	4
Regularization parameter	2
Tree depth	4
Learning rate	0.15
The Model of Iterative Tree	Gbtree
Loss function	Multi: Softmax

Different models have different evaluation indicators. In the regression model, the evaluation indicators include Mean Absolute Error (MAE), Mean Square Error (MSE), Root Mean Square Error (RMSE), and coefficient of determination (R^2^), as shown in Eqs (1)–(3):

$$MAE=\frac{1}{N}\sum_{i=1}^{N}\left|y_{i}-\hat{y_{i}}\right|$$ (1)

$$MSE=\frac{1}{N}\sum_{i=1}^{N}{\left(y_{i}-\hat{y_{i}}\right)}^{2}$$ (2)

$$RMSE=\sqrt{\frac{1}{N}\sum_{i=1}^{N}{\left(y_{i}-\hat{y_{i}}\right)}^{2}}$$ (3)

In Eq (3), $\hat{y_{i}}$ represents the predicted value. y_i_ represents the true value, i = 1, 2, 3,…, N.

The parameters of the model are learned from the training set. The specific strategy is to define a loss function for the model, also known as the risk function. The loss function is the difference between the predicted result and the true value of each sample [26]. It is assumed that the training set is $D=\{(x_{1},y_{1}),(x_{2},y_{2}),(x_{3},y_{4}),\ldots ,(x_{n},y_{n})\}$ and *n* is the sample size. $x_{i}=(x_{i}^{\left(1\right)},x_{i}^{\left(2\right)},x_{i}^{\left(3\right)},\ldots ,x_{i}^{\left(m\right)})$. m is the number of total features, and *i* = 1,2,3,…,*n*. If the model trained by the training set is *f*, for example (*x*_*i*_, *y*_*i*_), the predicted result using the model f is *f*(*x*). *L*(*y*_*i*_, *f*(*x*_*i*_)) represents the error degree between *y*_*i*_ and *f*(*x*_*i*_), that is, the loss function. Common loss functions are as follows.

(1) 0–1 loss function

$$L(y_{i},f(x_{i}))=\{\begin{array}{c} 1\;y_{i}\neq f(x_{i})\\ 0\;y_{i}=f(x_{i}) \end{array}$$ (4)

(2) Absolute loss function

$$L(y_{i},f(x_{i}))=|y_{i}-f(x_{i})|$$ (5)

(3) Quadratic loss function

$$L(y_{i},f(x_{i}))={\left(y_{i}-f(x_{i})\right)}^{2}$$ (6)

(4) Exponential loss function

$$L(y_{i},f(x_{i}))=e^{-y_{i}f(x_{i})}$$ (7)

(5) Logarithmic loss function

$$L(y_{i},P(y_{i}{|x}_{i}))=-logP(y_{i}{|x}_{i})$$ (8)

The loss function is for a single model. For *n* samples in the training set, the total loss function of the obtained model is the sum of the loss values of a single sample. With the quadratic loss function as an example, the total loss function of training set *D* is:

$$L(\beta ,b)=\sum_{i=1}^{n}{\left(y_{i}-f(x_{i})\right)}^{2}$$ (9)

*β* refers to the *n*-dimension parameter vector of the model *f*, and *b* is the set threshold value. The ultimate goal is to minimize the total loss function *L*(*β*, *b*). The problem of solving the model’s unknown parameters will be converted into the solution equation.


$$minL(\beta ,b)=\sum_{i=1}^{n}{\left(y_{i}-f(x_{i})\right)}^{2}$$ (10)

By defining the loss function and adopting the strategy of minimizing the loss function, the parameter-solving problem is transformed into an optimization problem. There are many methods to solve optimization problems, such as the gradient descent method, the damped Newton method, and Quasi-Newton Methods [27].

**Fig 8:**
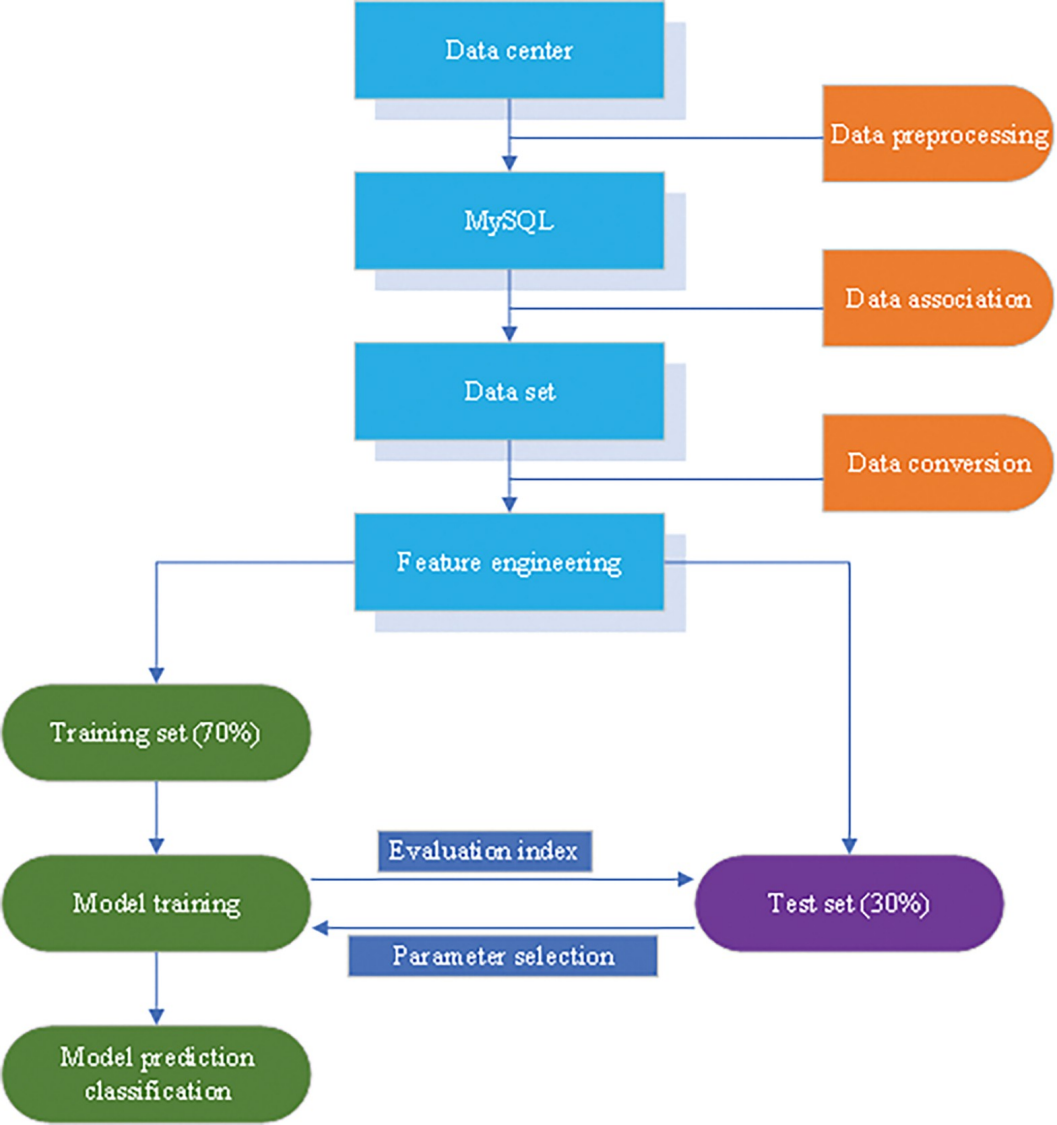
Training implementation process of the model.

Finally, based on the established data platform, this paper constructs a dataset based on highly shared data. The XGBoost classification algorithm is used to classify data mining automatically. The basic implementation process of model training is shown in Fig 8:

The collected data is pre-processed through the data center, and MySQL is used for data association. As a result, the relevant parameters of the dataset are set and formed into a dataset. The organized data is transformed and analyzed through feature engineering, and the training set and dataset are operated on separately. The experiment trains the model in the training set and obtains predictive classification, comparing the feasibility of the model for data mining.

## 4 Model design

**Fig 9:**
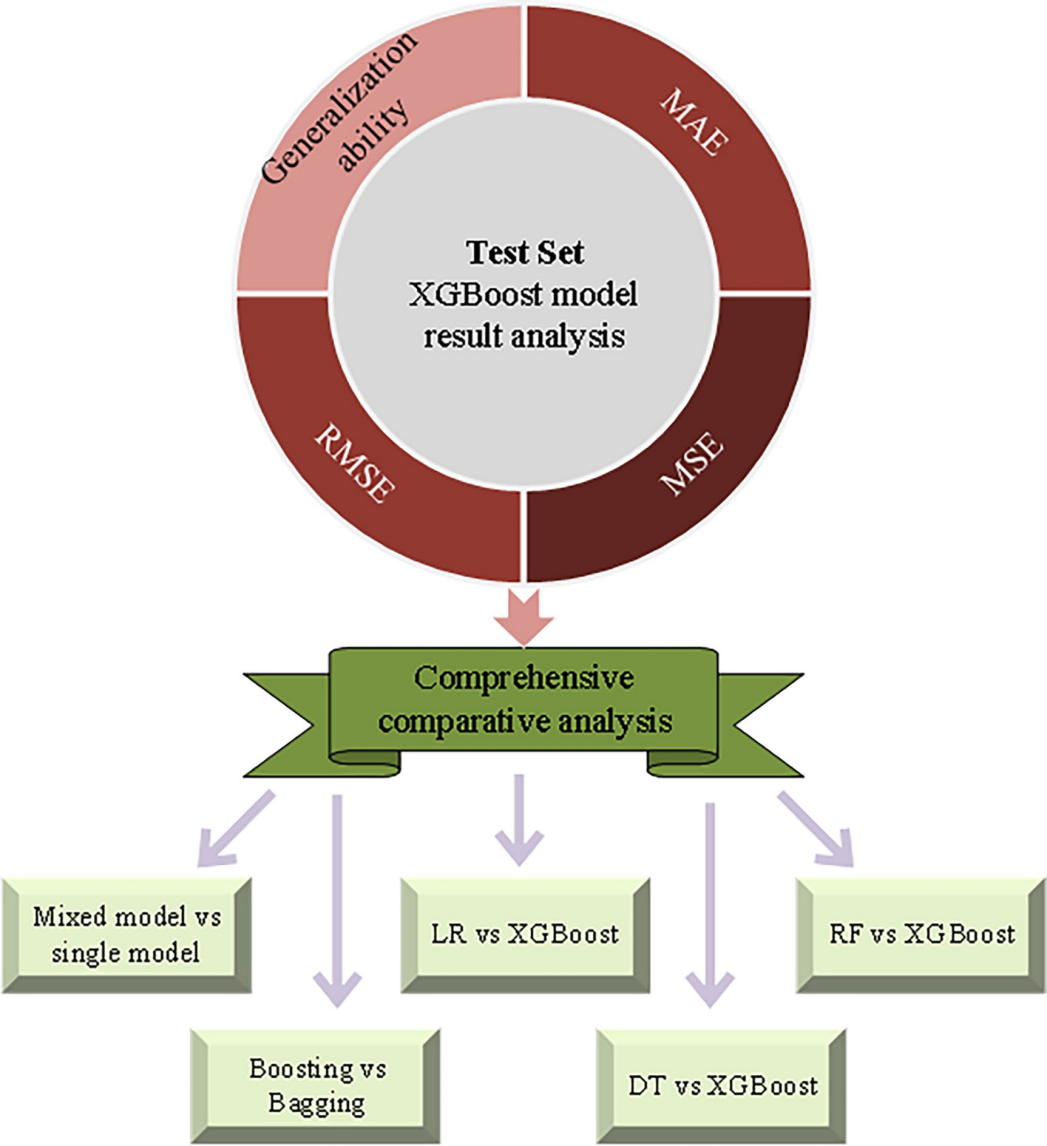
Evaluation architecture of XGBoost algorithm model (RF: Random forest algorithm; LR: Logical regression algorithm; DT: Decision tree algorithm).

Sales forecasting is an important part of business operations and is very important to the development of enterprises. As one of the classic algorithms in ML, XGBoost can combine several weak learners into a strong learning model and support parallel operation. It has shown good prediction performance in various prediction problems [28]. Hence, it is selected to build a financial enterprise sales forecast model. Fig 9 displays the model’s evaluation architecture.

**Fig 10:**
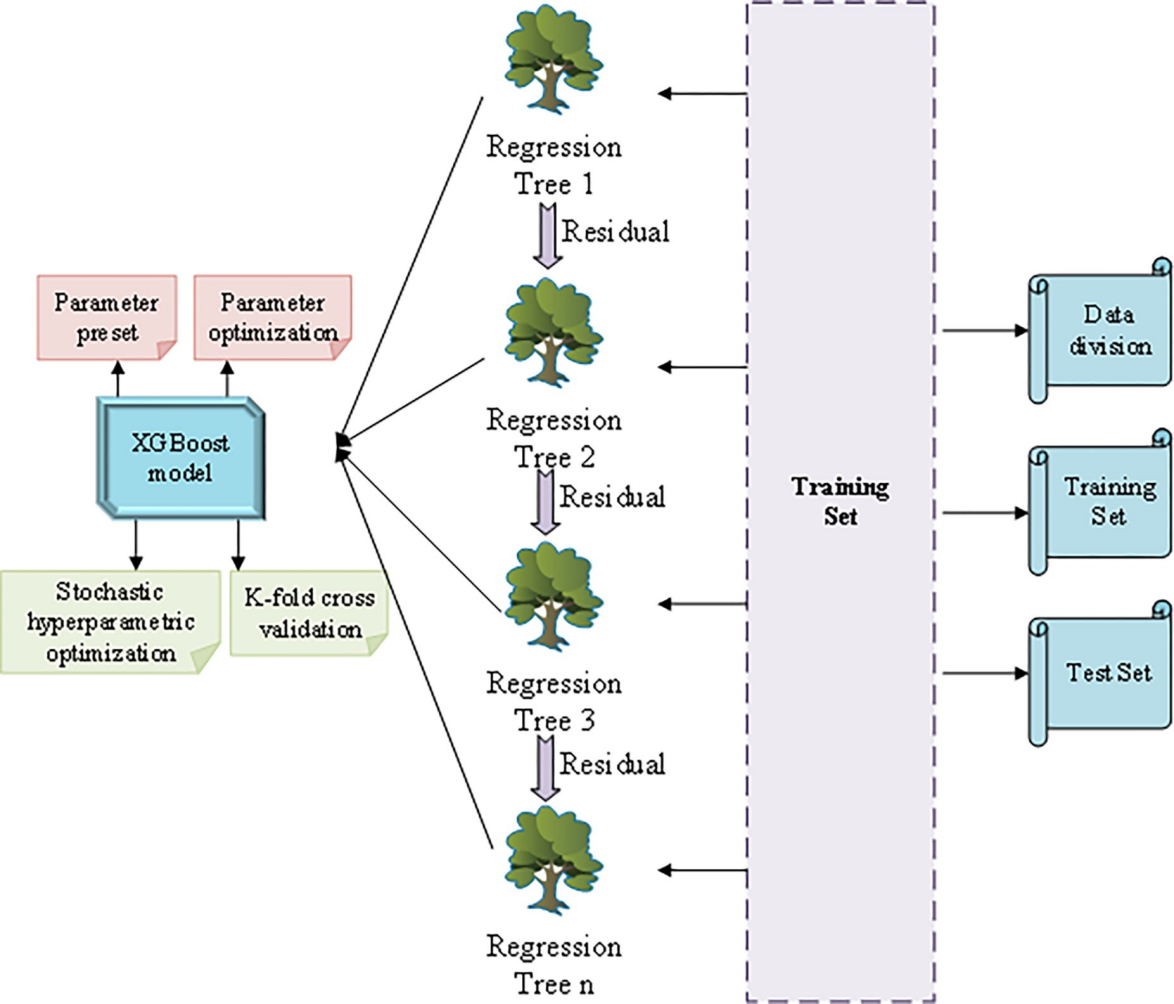
XGBoost algorithm training process.

The construction of an enterprise sales forecast model includes four parts: indicator design, data preparation, model training, and model evaluation. Enterprises must integrate different factors and design a reasonable sales forecasting indicator system when carrying out sales forecasting. Fig 10 illustrates the model training process.

This exploration draws on the worldwide relevant literature about the design of sales forecast indicators. Then, combining qualitative and quantitative methods, the prediction index system reflecting the macroeconomic environment, enterprise marketing, and financial activities is determined. Data are the basis of prediction. When establishing the sales forecast model, according to the designed indicator system, the data related to sales are collected from the internal database and external network environment of the enterprise through structured query language (SQL), crawler and other technologies. These data finally form the original data of the sales forecast model. According to different data sources, it is mainly divided into three aspects: BOSS, business, and network side. These data form the big data aggregation information base [29]. Additionally, each information source and its internal information storage layer are also different. Among them, network signaling data is mainly user basic data dimension table, including terminal dimension table, regional dimension table, music dimension table, and other dimension tables. BOSS mainly refers to the service data width table, including the signaling, the basic user information, and the specific service. Other business platforms are mainly marketing data pools, including marketing activities, activity effect data, active operation numbers, and recharging data [30]. These three types of data sources form a big data aggregation information base, including all user information such as consumption, business subscription relationship, terminal, online behavior, mobile phone action, etc. The user data covered are shown in Table 2:

**Table 2:** Customer information database.

User population	Scope Representative	Number of areas	Identification quantity
Business district	Shopping crowd	1	12,000
School	College student	50	65,000
Metro	Floating population	1	280,000
Cinema	Audience	8	18,000
Industrial Park	Worker	70	650,000

**Fig 11:**
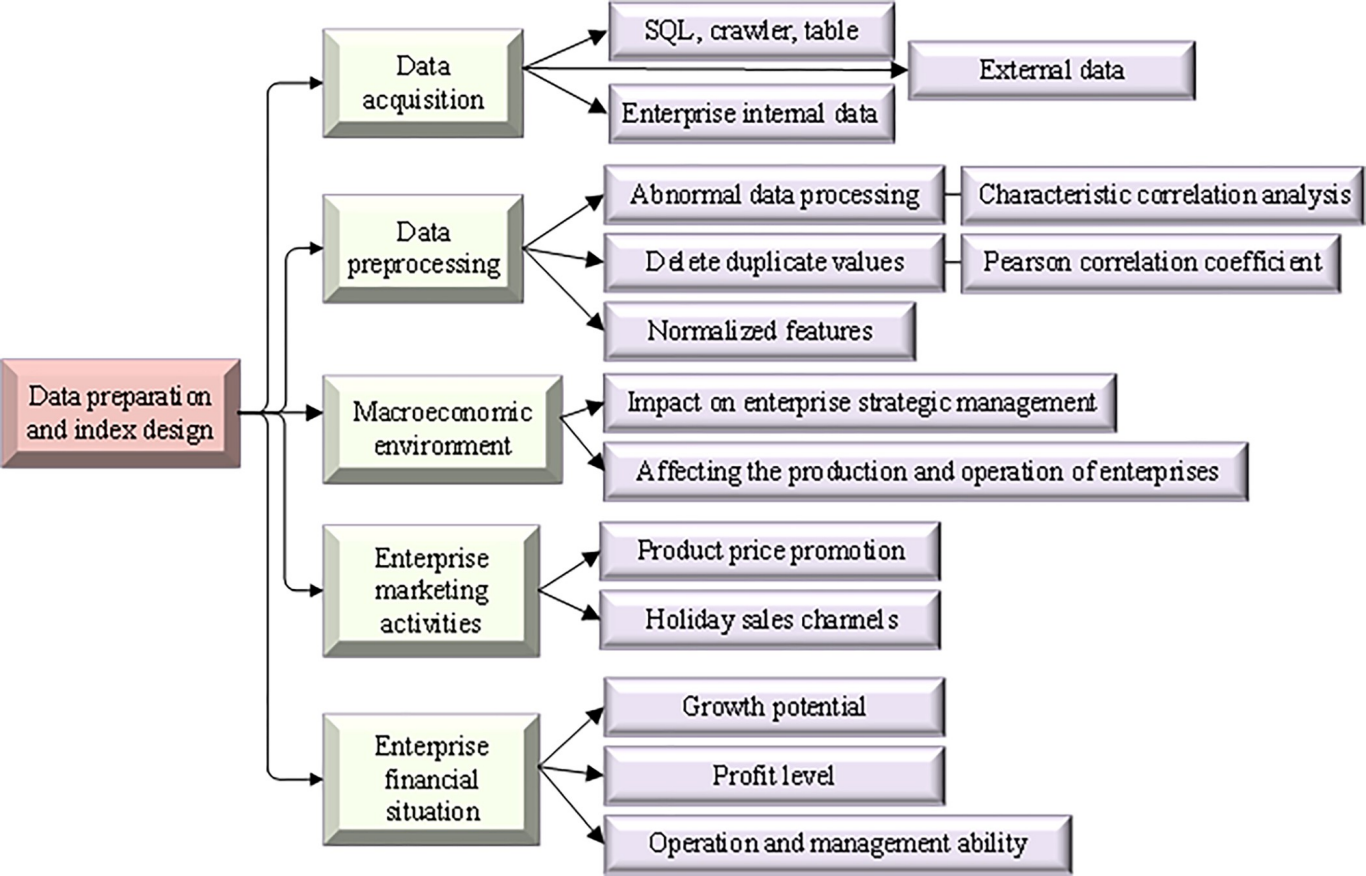
Data preparation and indicator design process of XGBoost model.

The interactive signalling between the user and the base station is used to identify social attributes and life circle. The identified users are divided into five categories: business district, industrial park, school, subway, and cinema. Each type of customer will form a model and file it. The data type in the original data is not uniform, and the data also contain a lot of noisy data. Hence, the original data should be pre-processed, including classification feature digitization, and abnormal data processing [31]. Fig 11 shows the process of model data preparation and indicator design.

Before using the XGBoost algorithm to train the sales forecast model of chain enterprises, the processed data need to be segmented. The dataset is classified into the test set and training set. The model is trained on the training set, and the model performance is evaluated on the test set. During model training, parameters need to be set and optimized [32]. Due to the large data span, target and characteristic variables are normalized. The adopted normalization calculation is shown in Eq (11):

$$X=(X-X_{min})/(X_{max}-X_{min})$$ (11)

In Eq (11), X refers to the characteristic vector.

This exploration uses the random search for parameter optimization to reduce time cost and avoid "dimension disaster". After model training, the model’s generalization ability needs to be evaluated. This exploration selects MAE, MSE, RMSE, and other indicators for evaluation. Besides, the sales forecast results under the XGBoost algorithm are compared with those under different algorithms to test the model’s superiority. This realizes the comparative analysis of the hybrid model and the single model, as well as the comparative analysis of the hybrid model based on boosting integration method and the hybrid model based on bagging integration.

Sales activities are a crucial part of enterprise production, operation, and management activities, which need to consider the macroeconomic environment of the market fully. In this regard, this exploration designs three indicators to reflect the macroeconomic environment: regional gross domestic product (GDP), per capita disposable income of all residents, and consumer price index. Table 3 shows the design of the indicators.

**Table 3:** Design of macro market indicators.

Serial number	A	B	C
Indicators	MAE	MSE	RMSE
Variable type	Data	Data	Data
Notes	Quarterly total value/3 (calculated by month)	Quarterly total value/3 (calculated by month)	Reflect the consumer price sub-index of the region

Market environment changes will greatly affect enterprises’ production, operation, management, and other activities. The macroeconomic environment has a direct impact on the strategic management of enterprises. Strategic management determines the future development direction of the enterprise and is the basic need to meet sustainable operations [33]. When formulating the strategy, the management should analyze the internal conditions of the enterprise and fully consider the macroeconomic environment. When the macroeconomic situation is good, and the enterprise’s conditions permit, it can expand its business scope, including increasing investment in existing products, or introducing new products, to increase its sales revenue and enhance its competitiveness.

## 5 Results and discussion

### 5.1 XGBoost algorithm detection

**Fig 12:**
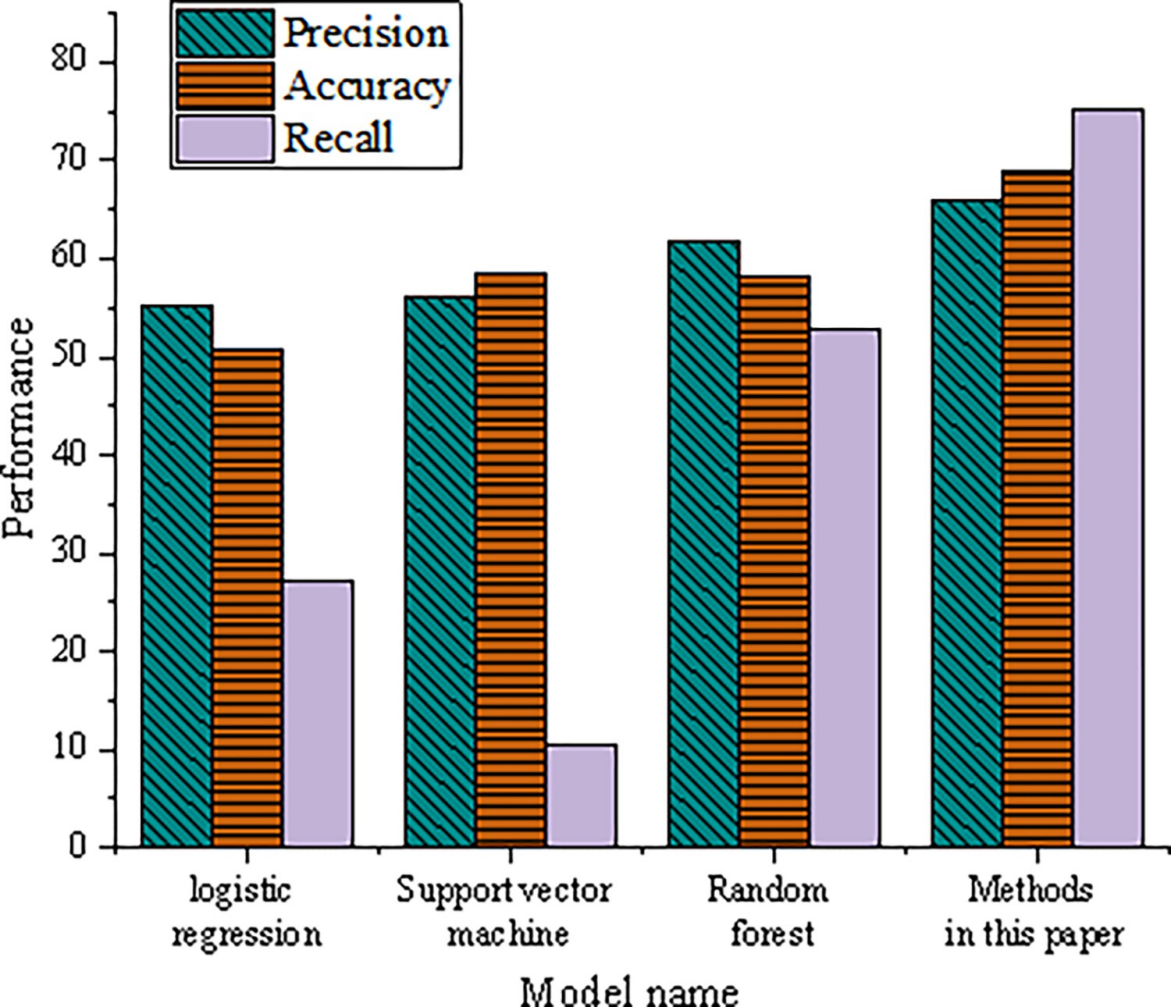
Comparison of the performance of four algorithms.

In order to confirm whether the method used in the model construction is the most effective, this exploration also compares the XGBoost method with the other four commonly used classification algorithms. It mainly selects three methods: LR, support vector machine (SVM), and RF. The optimal model is selected through the evaluation indicators’ final calculated value. Fig 12 shows its evaluation indicators.

The results of the comparative analysis of various indicators show that the methods used are higher than the other three methods in precision, accuracy, and recall, so the XGBoost algorithm has the best prediction effect.

The XGBoost algorithm model is used for model testing in the test set. The sales volume of the enterprise is predicted through experiments, as shown in Table 4:

**Table 4:** Prediction results of XGBoost algorithm.

	A	B	C	D	Precision rate
A	241	7	1		97%
B	20	293	18		89%
C		9	420	17	94%
D		5	61	3309	98%
Total					94%

In Table 2, the overall prediction performance of the XGBoost algorithm is at a medium to a high level. The accuracy of predicting annual sales revenue for enterprises can reach 94%, which is of great help for proposing marketing strategies. Additionally, this paper combines multiple linear regression, decision tree, random forest algorithm, and XGBoost algorithm to compare enterprise sales forecasts. Experiments show that the prediction performance of the hybrid model is better than that of the single model, as shown in Table 5:

**Table 5:** Comparison of enterprise sales forecast models.

Model		MAE	MSE	RMSE
Single model	Decision tree	0.0012	0.0001	0.0048
	Multiple linear regression	0.0037	0.0001	0.0104
Hybrid model	XGBoost	0.0007	0.0001	0.0025
	Random forest algorithm	0.0009	0.0001	0.0031

In Table 5, the prediction performance of the hybrid model is better than that of the single model. The accuracy of the enterprise sales forecasting model based on XGBoost and the random forest is higher than that based on multiple linear regression and decision trees. The XGBoost sales forecast model has relatively high accuracy, and the error is acceptable. The forecast is effective. This model can help enterprises solve the problem of the sales forecast.

This exploration tests the differences between the top 7 features that affect prediction results after the model training. It tests whether there are significant differences in the impact of these seven features on the results. Among them, the stock customers holding activated credit cards are the experimental group, and the inactive customer samples are the control group. In the resulting test, the chi-square test method is adopted. The chi-square tests the goodness of fit between characteristic variables and expected values based on chi-square distance analysis. Table 6 presents the results of the final inspection.

**Table 6:** Significance test of the difference between the results of important characteristics.

Characteristic variable	Method of use	Return variance	Unit nature	Industry category	Position	Average amount	Annual fee code
Chi-square value	290	12.7	254	287	68.2	245	237
Significance	0.0002	0.0006	0.0001	0.0008	0.0007	0.0006	0.0009

The test results show that the significance p values of the features included in the test are all less than 0.001, indicating that there is indeed quite a significant difference between these features and the results of activation or not. The following is an attribution analysis based on the three dimensions of the account activation indicator system.

### 5.2 Analysis of the impact of marketing strategy on customers

**Fig 13:**
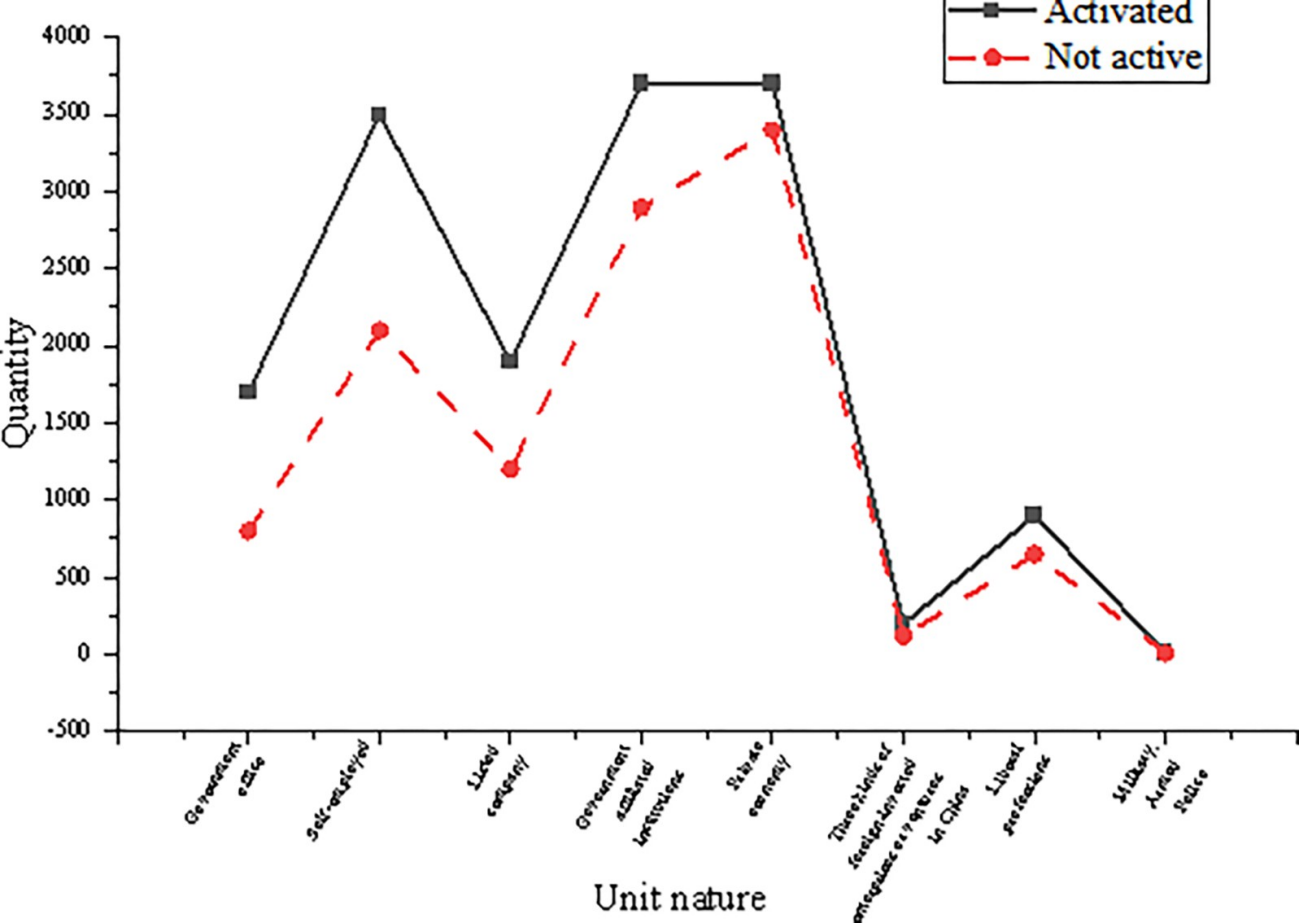
Relationship between the nature of different enterprises and customer consumption.

From the analysis of the relationship between the customer basic information dimension and customer activation, among the customer’s basic information dimensions, the most influential characteristics of the forecast model are the customer’s company nature and the customer’s position. Fig 13 shows the relationship between the nature of different units of customer work and whether the customer is activated.

**Fig 14:**
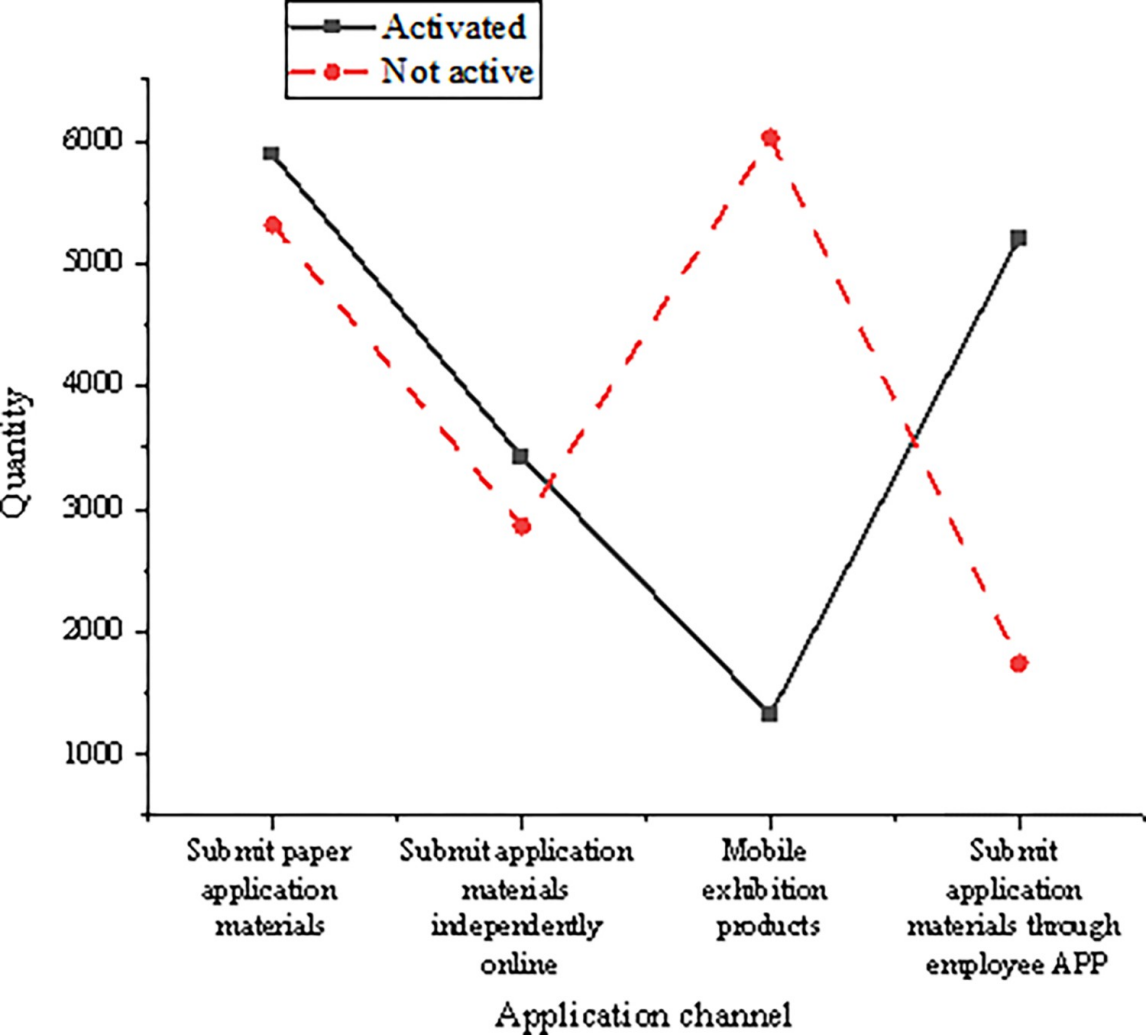
Distribution diagram of customers’ application in different channels of the enterprise.

When the customers are government agencies and individual businesses, the number of activated customers is far more than that of inactive customers. Government agency customers are of high quality. Hence, banks will generally allocate good card opening and activation rights when marketing their credit cards, such as some exquisite gifts that promote their card opening and activation. However, individual businesses often need a large amount of capital to carry out a turnover in their daily operations. Therefore, opening and activating credit cards and carrying out overdraft consumption in advance can help alleviate the financial pressure encountered in the daily operation process, which is also an incentive to open cards. Fig 14 shows the distribution of customers’ applications in different enterprise channels.

Customers who submit application materials through employee software and receive cards through employee handheld mobile exhibition equipment channels have higher activation. With the progress of technology, under the condition of controllable risk, the bank credit card customer manager has been supported to market customers on the spot and issue the card immediately after passing the on-site risk rating score by using the customer’s certificate and social security payment or housing provident fund image data. Currently, the customer manager can use some small gift rights on the site to stimulate the customer to activate and swipe the card. Compared with the traditional way that the back office center must manually approve paper application materials, and the cards need to be sent out while submitting application materials, telephone notification for card opening and other links greatly shorten the card issuing cycle. Moreover, the activation effect of face-to-face marketing is better than that of off-site marketing.

## 5.3 Discussion

Based on the highly shared data center of a vocational and technical college, Wu explored the application of the big data mining classification algorithm XGBoost in student management. Through the data center, students’ multi-dimensional features in various business systems are extracted as algorithmic data sources, providing a solid foundation for algorithmic models’ high accuracy and interpretability. Research has confirmed that using big data mining classification algorithms to utilize school’s big data properly can help schools manage students better [34]. Luo designed an evaluation system for Mergers and Acquisitions (M&A) risk indicators affecting state-owned listed companies and used Python to crawl web pages and text data. They applied machine learning XGBoost algorithm to construct an early warning model to achieve risk measurement, monitoring, early warning, and management. This result is compared with other classic models in experiments to evaluate the warning effect. Finally, the multiple linear regression model was used to study the significant factors of M&A risk. The empirical results indicate that the prediction accuracy based on the XGBoost algorithm is 80%, and it performs best among all models [35]. The above studies have confirmed that XGBoost has good data mining and analysis ability and has the characteristics of fast training speed, strong fitting ability, and can better avoid overfitting. This algorithm has become one of the most used algorithms in data mining and machine learning.

The cost of establishing the sales forecast system is classified into explicit cost and implicit cost. Explicit cost refers to various expenses that can be accurately quantified during the construction of an enterprise sales forecasting system. The implicit cost is quite secretive and difficult or impossible to quantify. The cost of building a sales forecasting system for chain enterprises can be divided into three stages. The first is the preparation stage. The explicit cost is the hardware equipment and software purchased for developing the sales forecasting system and server rental, consulting service, and research fees. Implicit costs include inefficient development, excessive expenses, and other costs arising from the selection of hardware equipment and servers that do not match the development of the enterprise due to information asymmetry, as well as the opportunity cost of related expenditures for other investments. The second is the implementation stage. Explicit costs include labor costs and software implementation costs. The labor cost refers to the expenses of salaries, bonuses, welfare, and insurance of technical personnel. The software implementation cost refers to other expenses paid to the software supplier during the software launch process, such as the incremental cost of introducing the latest software system. In Li’s research, the implicit cost mainly includes three aspects. The first is the human cost incurred by the risk control department and other departments before the system construction to help complete the sorting of the business process. The second is the implicit cost caused by the difficulty of communication between technical and financial personnel. This cost is caused by the fact that the technical personnel does not understand finance and the financial personnel does not understand technology. Moreover, the developed sales forecast system must consider the use of enterprise financial personnel comprehensively and the budget management of finance and other issues [36]. The third is the maintenance stage. Explicit costs include the salaries of operation and maintenance personnel and the related expenses incurred in continuously updating platform technology. The implicit cost is mainly the continuation of the implicit cost in the preparation and implementation stages. This is similar to the discussion results of this exploration.

## 6 Conclusion

From the marketing strategy perspective, this exploration integrates computer data mining and the XGBoost algorithm. Based on the analysis of the factors influencing the marketing of chain enterprises and customer consumption, the prediction index system is constructed from the market environment, marketing activities, and financial situation. It puts forward the realization form of introducing data mining into marketing and clarifies the theoretical basis and research direction. This method proposed is compared with the other three methods. The research results show that: (1) the accuracy and recall rate of the method proposed is 65.83% and 75.17%, respectively, which are greater than the other three methods. It can prove that the algorithm used here is optimal. (2) In the difference significance test of feature results, the p values of significance are all less than 0.001, indicating that there is indeed quite a significant difference between the feature and the result of whether the card is activated or not. (3) After implementing the proposed marketing strategy, the card opening and use of government agencies and individual business customers have achieved remarkable results. It can help customers alleviate the financial pressure in daily operations and urge them to buy products. This paper can contribute to future enterprise marketing. The XGBoost-based enterprise sales prediction model has high prediction accuracy and can help enterprises make corresponding marketing strategies. (2) The improvement of the connection rate and stickiness between enterprises and customers can have a huge driving effect on overall customer marketing.

This exploration needs to be improved in the following aspects. (1) The sales forecast in the marketing process is affected by various factors, and the sales of different goods in different enterprises are different. Due to the availability of financial-related data on the research object, the design of financial-related indicators is not comprehensive. (2) Although XGBoost performs well in prediction problems, it also has some defects. For example, determining the number of regression trees, learning rate, sampling rate, and other parameters in the algorithm lack effective theoretical support. In the future, grid search or other methods can be applied to determine better parameter values.

## Supporting information

S1 Data (ZIP)
